# Reflectivity and spectral shift from laser plasmas generated by high-contrast, high-intensity KrF laser pulses

**DOI:** 10.1098/rsta.2020.0043

**Published:** 2020-10-12

**Authors:** Zs. Kovács, K. Bali, B. Gilicze, S. Szatmári, I. B. Földes

**Affiliations:** 1 Department of High Energy Experimental Particle and Heavy Ion Physics, Wigner Research Centre for Physics, H-1121 Budapest, Hungary; 2 Department of Experimental Physics, Interdisciplinary Excellence Centre, University of Szeged, H-6720 Szeged, Hungary; 3 Department of Photonics and Laser Research, Interdisciplinary Excellence Centre, University of Szeged, H-6720 Szeged, Hungary

**Keywords:** laser plasma, ultrashort pulse, ultraviolet, reflection

## Abstract

The energy and spectrum of the reflected 248 nm radiation are studied from solid targets up to 1.15 × 10^18^ W cm^−2^ intensity. The experiments used the 700 fs directly amplified pulses of the KrF system which was cleaned from prepulses with the new Fourier-filtering method providing 12 orders of magnitude temporal contrast. Increasing the intensity from 10^15^ W cm^−2^ results in increasing absorption up to more than 90% above 10^18^ W cm^−2^. This is accompanied by increasing x-ray conversion exhibiting a less steep power law dependence for low-Z matter than for gold. Strong blue shift of the reflected radiation from the backward propagating plasma was observed. It is shown that in the case of KrF laser pulses of highest contrast, vacuum heating can be one of the dominant absorption mechanisms.

This article is part of a discussion meeting issue ‘Prospects for high gain inertial fusion energy (part 1)’.

## 1. Introduction

KrF lasers have been and even presently are regarded as possible alternative laser drivers for inertial confinement fusion (ICF) [1]. The short wavelength and the high beam quality make them attractive not only as a driver but also as a fast ignitor [2] because the electrons will not be accelerated much above 1 MeV even in the nonlinear interactions with the short pulse. Thus, the energy of the accelerated electrons can be well absorbed in the fuel. A fast ignitor scheme based on KrF lasers was also suggested in which the same amplifiers can be used for both the driver and the ignitor [3]. KrF lasers are also possible candidates for the shock ignition scenario of inertial fusion [4,5]. As recent interest arose in direct drive schemes, intense studies of excimer lasers such as KrF and even ArF (with even shorter wavelength) for fusion [6] started again.

The basic phenomenon to be studied in laser–plasma interactions with UV pulses is the reflectivity, i.e. the absorption. The investigation of the reflectivity, the spectrum of the reflected radiation and the x-ray conversion in plasmas generated on the surfaces of solid targets by ultrashort laser pulses have been the subjects of great interest in the last three decades. The relevance of these studies to ICF, and especially to fast ignition, was discussed by Myatt *et al.* [7], in which not only absorption but x-ray conversion and fast electrons were also investigated. Several processes contribute to the possible observations; the results depend not only on the wavelength, intensity and pulse duration of the pump pulses, but also on their temporal profile [8], especially on the intensity ratio of the ultrashort main pump pulse to the initial prepulse. It is proven that a prepulse generated preplasma modifies the initial boundary conditions and then even the absorption process. Detailed investigations were published on the effect of intensity contrast on absorption of the laser pulse [9] even recently. Most of these studies were carried out by infrared pulses from solid-state lasers.

The blue Doppler shift from the expanding plasmas had already been observed in 1988 and interpreted therein by thermal expansion [10]. Detailed investigations [11] showed the slowing down of the expansion with increasing intensity and demonstrated the importance of ponderomotive effects. With the available higher laser intensities, it was soon shown that in cases where the laser intensity becomes relativistic, the red spectral shift of the reflected radiation becomes dominant [12], which was later attributed to light pressure and hole boring [13]. Detailed theoretical analysis was carried out by Wilks & Kruer [14] who considered several absorption mechanisms such as collisional absorption, resonance and Brunel absorption [15] for the nonrelativistic regime and **j × B** heating for the relativistic case. Recently, the calculations were extended toward the interactions with petawatt lasers taking into account relativistic hydrodynamic motion as well [16]. Detailed studies were carried out in the ultrarelativistic regime showing increasing absorption with increasing intensity [17] and the red shift from the inward propagating hole boring, which showed a saturation behaviour for 10^20^ W cm^−2^ intensity [18]. Recent experimental and theoretical studies up to 2 × 10^21^ W cm^−2^ pump intensity even investigated the effect of target thickness as connected to radiation pressure effects [19]. A comprehensive review of absorption processes, together with different acceleration mechanisms such as stochastic heating and betatron acceleration, was published recently [20]. As discussed in this paper, absorption processes depend strongly on the initial conditions; i.e. the results also depend on the conditions of the eventual preplasmas, and thus, on the contrast of the pump beam. These effects were recently investigated experimentally by Singh *et al.* [9].

The investigations with short-pulse KrF lasers, offering pulses in ultraviolet, are quite special. Due to the short wavelength of the radiation one expects a deeper penetration, and consequently higher absorption. It is a unique feature of KrF excimer systems that direct amplification is used after frequency conversion of a seed pulse, where the temporal pedestal of the 248 nm pulses originate only from the amplified spontaneous emission (ASE) of the amplifiers. For this reason—in contrast with solid-state CPA systems—no significant picosecond pedestals are present, just the nanosecond duration ASE pulse of homogenous temporal profile. One of the first detailed short-pulse absorption measurements using a KrF laser were carried out by Fedosejevs *et al.* [21]. The interpretation therein was, however, phenomenological, based on the Drude model. Detailed investigations were carried out with the available higher intensities by Teubner *et al.* [22]. Besides the detailed absorption measurements hot electron production and x-ray conversion were also studied, in the latter case observing a power law dependence on the absorbed intensity [23]. Especially interesting is the estimation of the plasma acceleration in the paper of Sauerbrey [24]. A very high acceleration was obtained using KrF laser pulses of relatively low ASE pedestal, and in the interpretation of the experimental data even the chirp of the pulse was taken into account. Surprisingly—for the highest, but still nonrelativistic laser intensities—not only blue shift was reported from the expanding plasma, but also a red shift.

Due to the short wavelength of the KrF radiation, the relativistic limit for the focused intensity is 16 times higher than that for the 1 µm wavelength. In our case, the highest intensity was 1.15 × 10^18^ W cm^−2^ which is still nonrelativistic. On the other hand, a negative consequence of the short wavelength is the strong ASE. The intensity of the ASE develops rapidly even if the amplifiers are used in an off-axis geometry, and its focusability is much worse than that of the main pulse. Mass spectrometry shows that a significant amount of ions are produced due to photoabsorption and photoionization even if the ASE intensity is as low as 10^7^ W cm^−2^ [25]. Although the energy contrast can be improved by spatial filtering, special efforts are needed to improve the intensity contrast. Although plasma mirrors are applicable [26] for KrF systems, finally the so-called Fourier-filtering method [27] proved to be appropriate for simultaneous spatial and temporal filtering of the KrF radiation. Integrating this new filtering technique intensity contrast up to 10^12^ was realized for the high-intensity pulses and applied in the present experiments [28].

These improvements of the parameters of the KrF laser systems allowed us to carry out investigations on the reflectivity of laser light from solid targets up to 1.15 × 10^18^ W cm^−2^ intensity with an intensity contrast of 10^12^. The spectrum of the reflected radiation was also investigated in the full range of interest. Earlier plasma mirror experiments were optimized to obtain contrast improvement by minimizing losses. In those cases, the reflectivity was measured to saturate at 10^15^ W cm^−2^, i.e. the experiments of Gilicze *et al.* [26] were carried out at lower intensities. Here, investigations for higher intensities are reported together with the study of conversion to x-rays. The motivation of these experiments lies in the fact that a broad range of nonrelativistic intensity could be covered, which allows us to obtain a clear picture of these phenomena. In comparison, measurements were also carried out with pulses of moderate contrast of 5.5 × 10^5^, when the prepulse intensity was high enough to generate preplasmas.

## 2. Methods

**Figure 1.**
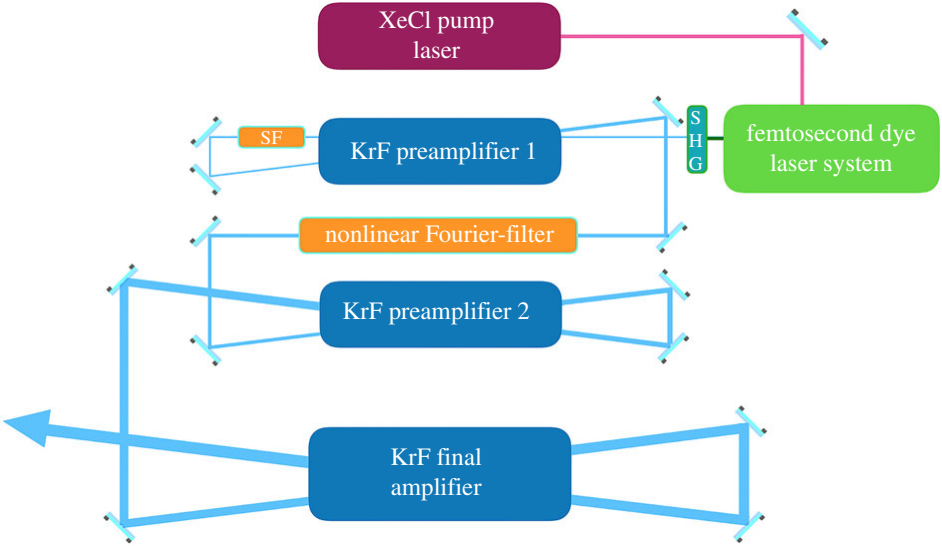
Schematic of the laser system. SHG is the second harmonic generation of the seed pulse, SF is the spatial filter.

Our laser system used in the following experiments is an updated version of the Szatmári-type hybrid-excimer laser system [29]. The KrF system is based on the amplification of a frequency doubled output of a 500 fs distributed feedback dye laser oscillator-amplifier setup. The pulse was amplified in a chain of discharge pumped KrF amplifiers of increasing cross-section. The amplification is realized in two-pass off-axis geometry [29] in each stage. After the first pass in the first amplifier, the pulse is sent through a pre-imaging system, comprised of a two lens telescope and a conventional spatial filter. After the second pass, the nonlinear Fourier filter was integrated into the system [28]. The laser arrangement is illustrated in figure 1. The difference between the present arrangement and the one previously described [28] is that in the present case the polarization multiplexer was left out in order to preserve the direction of the original polarization. The final energy was 80 mJ and after propagating 4 m in filled air tubes the pulse duration was measured to be 700 fs FWHM for the sech^2^ pulse. Due to the large number of optics—as required by the temporal filter and by the windows of the three amplifiers—the polarization contrast of the p-polarized radiation decreased to 7 : 1, and the pulse inhibited a positive chirp of 3.5 × 10^−5^ fs^−2^ on the target.

The 4 cm × 4 cm flat-top beam was focused in a vacuum chamber (with a pressure of less than 10^−5^ mbar) by a SORL F/3, 30° off-axis parabolic mirror to a slightly elliptical spotsize of (1.85 ± 0.1) µm FWHM for the vertical axis and (1.95 ± 0.1) µm for the horizontal axis (corresponding to an approx. 2× diffraction limit). More than 70% of the laser energy was concentrated in the central lobe. Using the Fourier-filtering, the energy contrast was 80 : 1, but due to the longer pulse duration and the lower focusability of the ASE (its spot diameter was measured to be 1.56 mm) the intensity contrast surpassed 10^12^. As a comparison, experiments were also carried out without the Fourier filter when the contrast was reduced to 5.5 × 10^5^. In the following, ‘high contrast’ refers to the 10^12^ temporal contrast of intensity when the Fourier-filter is in use and ‘low contrast’ when not. To ensure optimum pulse-to-pulse energy stability, the laser was operated in a repetitive mode of 1 Hz, and single shots were selected by a shutter to hit the target.

The targets were vacuum evaporated boron- and gold-coated (500 nm thickness) float glass targets. They were found to be flat by a profilometer in the whole range of investigations, and the measured surface inhomogeneities of typically less than 50 nm were less than *λ*/5 of the wavelength applied. The angle of incidence of the pump beam was 45°, thus the maximum intensity on the target was 1.15 × 10^18^ W cm^−2^. In order to avoid debris on the focusing optics, a thin 1 mm thick suprasil quartz-plate was placed between the mirror and the target. This did not affect the quality of focusing and introduced no spectral change of the incident beam. For target positioning, an xyz translation stage was used (STANDA 133 373), with a step size of 1.25 µm. The stability of the target holder was sufficient because the deviation was only ± 2.5 µm in the direction of the beam across the sample of 4 cm. This is well within the 6 µm Rayleigh range. The intensity was varied using a variable diaphragm. For each aperture size, the focal spot was also measured and it was taken into account when defining the intensity. Note that the attenuation had no significant effect on the contrast. This method allowed us to vary the energy from 55 mJ to 0.8 mJ, corresponding to more than three orders of magnitude intensity span on the target.

The position of the focal plane was controlled by the intensity dependent x-ray yield using an IRD AXUV-100 silicon diode with a 2 µm-thick aluminium filter. The same diode was used to measure the intensity dependence of the x-ray radiation, too. After the interaction of the incident beam with the target, an f = 15 cm lens was used to collimate the specularly reflected beam and was sent either to a Gentec QE50LP-S-MB-D0 pyroelectric energy meter, or to a spectrometer of 0.027 nm spectral resolution.

## 3. Experimental results

**Figure 2.**
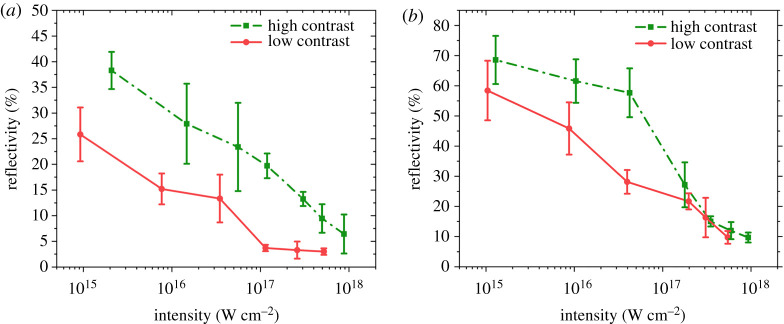
Measured reflectivity from (*a*) gold and (*b*) boron targets. The red circles (solid line) correspond to the case of low and the green squares (dotted line) the high-contrast case.

Reflectivity was measured after focusing the collimated beam onto gold (Au) and boron (B) targets, and the losses in the optics were taken into account. Since the reflected light had a contour of well-defined diameter, the scattering into a larger cone was excluded. The measured intensity dependence is shown in figure 2, showing similar behaviour for both Au and B. Following the expectations, in the low-contrast case, the reflectivity is lower in the whole range of intensity, i.e. the larger plasma absorbs more radiation.

Increasing the intensity from 10^15^ to 10^18^ W cm^−2^, the reflectivity shows a monotonic decrease. Note that former plasma mirror experiments performed with KrF lasers where increasing reflectivity was measured for the 45° angle of incidence showed saturation above 10^14^ W cm^−2^ and a strong decrease above 10^15^ W cm^−2^ intensity [30]. In the present work, however, the reflectivity drops below 10% above 10^18^ W cm^−2^, i.e. the absorption surpasses 90%. This tendency is similar to that found by Ziener *et al.* [31] for infrared pulses, who also observed an increase of absorption up to the same *Iλ^2^* value, i.e. up to several times 10^16^ W cm^−2^ infrared intensity. Our results can be compared with the recent results of Singh *et al.* [9], too. Their experiments were carried out with shorter (30 fs) pulses at intensities above 10^17^ W cm^−2^ using pulses of high, low and medium contrast. The lowest intensities there correspond to nearly the same *Iλ^2^* as the highest ones applied here, therefore it is not surprising that for the highest, 1.15 × 10^18^ W cm^−2^ intensity case we see similarly little difference between the high- and the low-contrast data. The difference is that absorption is significantly higher in our case using a KrF laser which is a probable consequence of the large penetration depth for the short wavelength as will be discussed later.

**Figure 3.**
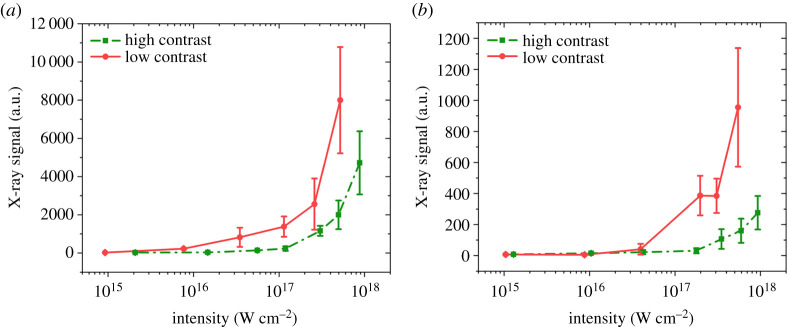
X-ray signals for (*a*) gold and (*b*) boron targets. The red circles (solid line) correspond to the case of low and the green squares (dotted line) to the high-contrast case.

The intensity dependence of x-ray emission is illustrated in figure 3 for the two targets both for the high- and low-contrast case. The low-contrast pulse clearly results in more x-ray emission in both cases. The data here are in arbitrary units but the two targets and the two cases are comparable. The high-Z material, namely gold, emits roughly an order of magnitude more x-rays than boron. Note that as a consequence of the Al filter used in front of the detector, in the case of B targets only the Bremsstrahlung emission is observed (as B has no lines above 1 keV); however, in the case of Au the M-band contribution may be significant. It is also salient that x-ray emission has a steep increase above 10^17^ W cm^−2^. Similarly to early experiments with KrF lasers [22] in the high-intensity range the measured x-ray intensity is fitted with a power law function, i.e. $I_{\text{x-ray}}=I_{\text{laser}}^{\gamma }$. The results are summarized in table 1. In both cases of Au and for the low-contrast case of B, the fitted *γ* values are equal to that of Teubner *et al.* [22] within the accuracy of the measurement. However, for boron targets heated by a high-contrast laser pulse the dependence is significantly less steep representing a case when only free-free transitions are detected for an initially steep, stepwise density profile.

**Table 1.** The fitted *γ* factor for high- and low-contrast Au and B target.

	*γ*_Au_	*γ*_B_
low contrast	1.68 ± 0.17	1.64 ± 0.17
high contrast	1.64 ± 0.17	1.3 ± 0.08

**Figure 4.**
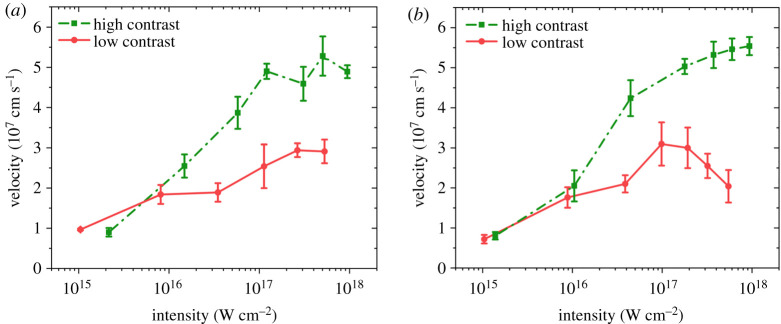
Velocities derived from the spectral shift of the laser pulse for (*a*) gold and (*b*) boron targets. The red circles (solid line) correspond to the case of low and the green squares (dotted line) to the high-contrast case.

The most interesting result is probably the observed spectral shift of the specularly reflected radiation. In the whole intensity range, i.e. from 10^15^ to 1.15 × 10^18^ W cm^−2^, a blue shift was observed for both target materials either for high- or for low-contrast pulses. Figure 4 illustrates the velocities derived from the measured spectral shift using the simple nonrelativistic Doppler law, i.e.

3.1 $$v=\frac{c}{2\cos\theta }\frac{\Delta \lambda }{\lambda }.$$

At a constant angle of incidence of *θ* = 45°, the velocity is proportional to the spectral shift. The data points are averaged over 5–8 shots for each intensity value. Whereas the blue shift and the corresponding velocity saturates above 10^17^ W cm^−2^ intensity for both targets in the case of low-contrast pulses (note that this tendency is more visible for boron), for the high-contrast case, the spectral shift is increasing until the upper limit of the intensity range of the present experiment. Nevertheless, even in these cases, the initiation of saturation is visible for the highest intensities. As seen in the figures, the highest blue shift observed was approximately 0.6 nm corresponding to a maximum velocity of 5–6 × 10^7^ cm s^−1^ and to a corresponding acceleration of approximately 1.5 × 10^18^ m s^−2^. The acceleration was derived by dividing the velocity by the half of the pulse duration. Note that these values are significantly higher than observed previously [24]. In those early experiments, however, the KrF laser system had a contrast of 10^8^–10^9^ only, i.e. significantly lower than in our present case; moreover, the maximum intensity presented here is four times higher. These are the possible reasons why the spectral shift and the corresponding velocity is much higher in the present case. It must be emphasized that the highest velocity values can only be obtained for the cleanest laser pulses, i.e. when the ASE prepulse intensity was well below 10^7^ W cm^−2^ for which the photoablation and photoionization can be disregarded [25]. In the whole intensity range, we were within a nonrelativistic regime for the UV radiation, and that is why only a blue shift was observed. The appearance of a red shift in a previous work [24] is rather a broadening of the reflected light (see figure 2b in [24]), probably caused by the photoablated material whose presence was supported by the relatively high prepulse intensity of approximately 10^8^ W cm^−2^. Detailed discussion of the phenomenon concerning the observed broadenings for low- and high-contrast radiation will be given elsewhere.

## 4. Discussion

Our experiments are the first ones which use temporally clean KrF pulses for high-power laser–plasma interaction studies using the new Fourier-filtering technique [28]. In the case of the very high contrast (which is significantly higher than the previous ones), our results are compared with the low-contrast interactions where significant preplasma could be present. The increasing absorption shows a similar tendency to that obtained with infrared lasers [31]. The higher absorption in our case can be attributed to the shorter wavelength of the KrF laser pulse and the consequently larger penetration depth. The results obtained herewith can also be compared with former ones obtained with short-pulse KrF radiation [21–23]. Following the initial low-intensity experiments [21], detailed absorption studies were carried out up to 2 × 10^17^ W cm^−2^ intensities and 1.5 × 10^10^ contrast [32]. In these studies, 70% maximum absorption was reached for Al and C, comparable to our finding at the same intensity in boron. Even higher, 80% absorption was reported in a subsequent paper [22] with a pronounced maximum at the 45° angle of incidence which was higher than expected from the simulations. Thus, our measured high absorption in boron is in good agreement with the earlier results. It can also be seen that the difference between absorption of very high- and very low-contrast pulses is small in boron, especially for intensities above 10^17^ W cm^−2^.

On the other hand, up to the highest intensities, absorption of pulses of poor contrast is significantly higher in gold. The photoionization rate is high for the metallic target and additionally the inverse Bremsstrahlung is larger for larger preplasmas. Note that the absorption at the highest intensity is very high, reaching approximately 95% in the presence of a significant preplasma. Simulations of early experiments [22] attribute these observations to collisional absorption up to 10^17^ W cm^−2^, whereas for even higher intensities, collisionless processes may be dominant [14]. We have to mention that the highest absorption with a remarkable maximum at the 45° angle of incidence [23] can be explained by resonant absorption [33], by an assumption of *λ*/L = 0.22 scalelength, which is typical for the contrast in those experiments. It is interesting that this scalelength for the same angle of incidence was found to be optimal for surface harmonics generation [34].

The most interesting case of x-ray conversion measurements is that of B. As an Al filter was applied in front of the x-ray diode, the line spectrum of B is fully blocked, the observed signals can be attributed to the Bremsstrahlung emission only—i.e. free-free transitions—whereas in Au a significant amount of harder, M-band radiation may also contribute. Similarly to Teubner *et al.* [23], we could also fit the observed x-ray energy with a power law function. The results seen in table 1 agree with those obtained therein within the experimental errors. However, in the case of the clean pulse, the dependence is less steep for B. The reason for this reason is connected to the steepest initial plasma profile, when free-free transitions are less important for the smaller plasma size. The Bremsstrahlung emission per unit volume is proportional to $n^{2}\surd T$, but the observed signal is proportional not only to the square of the density but also to the length of the plasma.

The Doppler shift and the corresponding velocity of the reflected spectrum at low intensities (i.e. up to 10^16^ W cm^−2^) show minor differences between the high- and low-contrast cases. With increasing intensities the spectral shift and the corresponding velocity saturate for the case of strong prepulse; however, for clean pulses, the spectral shift increases up to the highest level where weak signs of saturation become visible. This follows the expectations demonstrated in [12] that in cases of nonrelativistic intensities backward plasma propagation dominates. The observed 0.6 nm blue shift is significantly higher compared to that of Sauerbrey [24] and of our earlier observations [25]. In the present case, not only is the applied intensity higher, but—as seen e.g. in figure 4—the prepulse-free short pulse is more efficient in the acceleration either in the backward or in the forward direction [9].

In the following, the data obtained with high contrast will be discussed in detail.

The three main mechanisms of absorption in the nonrelativistic case were introduced in the 1990s [15], and they are also considered in the early works. Clearly, in an extended plasma, collisional absorption is dominant but what governs the interaction with the initially steep density profile? The interpretation of the above cited earlier experimental data included the effects of ponderomotive force, resonance absorption and vacuum heating. First of all, we must agree with the notes in [32] that even in the case of a subpicosecond KrF laser pulse of 10^18^ Wcm^−2^ intensity, it has a temporal wing of picosecond duration and of 10^14^ W cm^−2^ intensity. This might cause a 0.1*λ* scalelength by the time of the arrival of the main pulse. Definitely in detailed simulations the pulse shape must be taken into account. Herewith we carry out elementary considerations. As we are at intensities where even the Brunel mechanism might be present [35], the expansion velocity can be derived using the approximations of Wilks & Kruer [14]. The Brunel absorption can be given as

4.1 $$A=\frac{I_{\text{abs}}}{I_{0}}=8\frac{v_{\text{osc}}}{c}\text{si}{\text{n}}^{3}\theta ,$$

with

4.2 $$v_{\text{osc}}=\frac{e|E|}{m\omega }=\frac{e}{m\omega }\sqrt{\frac{8\pi I_{0}}{c}}.$$

The hot electron temperature derived from this oscillation velocity is approximately 11.5 keV for the maximum intensity in our case. The sound velocity corresponding to this temperature is approximately 7.3 × 10^7^ cm s^−1^. We must take into account also the light pressure which acts in the opposite direction. The velocity in the forward direction (i.e. opposite to the expansion) caused by the light pressure can be estimated as

4.3 $$v_{lp}=\frac{p}{\rho \delta }\tau \approx 1.1\times 10^{7}\text{cm}\;{\text{s}}^{-1},$$

with the assumption of p approximately 330 Mbar pressure, *ρ* is the density, *τ* = 350 fs half of the pulse duration (i.e. up to its peak) and the scalelength of *δ* = *λ*/5. The difference of the two velocities roughly equals the maximum velocity as illustrated in figure 4. Although the light pressure is not sufficient to compensate the backward acceleration, its role is probably not negligible. In a previous work [36], we pointed out that above 10^16^ W cm^−2^ the light pressure becomes comparable to the ablation pressure, thus it may contribute to the steepening of the plasma profile, and thus, to the macroscopic acceleration.

We have to note that the Brunel absorption itself gives approximately 80% absorption for 10^18^ W cm^−2^ intensity, thus it is probably one of the responsible mechanisms of our observations at the highest intensities. The real situation is probably more complicated. At relative low intensities, collisional absorption is probably still dominant. Resonance absorption and at the highest intensities Brunel absorption steps in at increasing intensity. Even in the case of the highest laser contrast, there might be a finite initial scalelength, consequently besides vacuum heating in that case even resonance absorption may be present.

## 5. Conclusion

The experiments on reflectivity and analysis of the reflected spectra are performed in laser plasmas produced by subpicosecond UV laser pulses of very high contrast. The achieved temporal contrast of 10^12^ is the result of the first time integrated Fourier-filtering method into the pump laser system. The interaction of high but still nonrelativistic intensities resulted in more than 90% absorption and the highest spectral blue shift from the expanding plasma. X-ray diagnostics show that the power law dependence of Bremsstrahlung emission is strongly dependent on the plasma size, resulting in a smaller exponent for the temporally clean laser pulse. The results obtained with greater than 10^18^ W cm^−2^ intensities can be explained by taking into account the ponderomotive force and Brunel absorption; however, the contribution of resonance absorption and even collisional absorption may also gain significance as the plasma expands from the initially steep profile.

## Data accessibility

This article has no additional data.
